# The role of chemical properties of the material deposited in nests of white stork in shaping enzymatic activity and fungal diversity

**DOI:** 10.1007/s11356-023-31383-x

**Published:** 2023-12-08

**Authors:** Ewa Błońska, Robert Jankowiak, Jarosław Lasota, Natalia Krzemińska, Adam Zbyryt, Michał Ciach

**Affiliations:** 1 Department of Ecology and Silviculture, Faculty of Forestry, University of Agriculture, Al. 29 Listopada 46, 31-425 Krakow, Poland; 2 Department of Forest Ecosystem Protection, University of Agriculture, 29 Listopada 46, 31-425 Krakow, Poland; 3 Faculty of Biology, University of Białystok, Ciołkowskiego 1J, 15-245, Białystok, Poland; 4 Department of Forest Biodiversity, Faculty of Forestry, University of Agriculture, Al. 29 Listopada 46, 31-425 Krakow, Poland

**Keywords:** C/N/P ratio, Fungal diversity, Ornithic, Soil ecology, Extracellular enzyme

## Abstract

Organic debris accumulated in bird nests creates a unique environment for organisms, including microbes. Built from various plant materials that are typically enriched by animal residues, bird nest favours the development of various fungal groups. The aim of this study was to investigate the chemical properties of the material deposited in the white stork *Ciconia ciconia* nests and the link between extracellular enzyme activity and the diversity and composition of culturable fungi. Our findings revealed low C/P and N/P ratio values in the nest materials, which indicate a high P availability. Nest material C/N/P ratio ranged from 67/8/1 to 438/33/1. Enzymatic activity strongly correlated with the content of carbon, nitrogen, and pH of the material deposited in the nests. A total of 2726 fungal isolates were obtained from the nests, from which 82 taxa were identified based on morphology and DNA sequence data. The study indicates that white stork nests are microhabitat characterised by diverse chemical and biochemical properties. We found relationship between the fungal richness and diversity and the C/P and N/P ratios of materials from the nests. Our study showed that culturable fungi occurred frequently in materials with high levels of C, N, and P, as well as high concentrations of base alkaline elements (Ca, Mg, and K).

## Introduction

Fungi exists in different types of environment and can survive across a wide range of pH and temperature (Rousk and Bååth 2011; Frąc et al. 2015) and they can help to stabilise organic matter (Treseder and Lennon 2015; Frąc et al. 2018). The accumulated organic debris present in constructed bird nests offers a unique environment for microbial assemblage (Baggott and Graeme-Cook 2002). Depending on the nest type (e.g. open-cup nests, tree cavities) or habitat location (e.g. aquatic or terrestrial), different fungal species become characteristic components of this specific microhabitat and play important roles in nutrient cycling (Apinis and Pugh 1967; Pugh and Evans 1970; Hubálek and Balat 1974; Hubálek and Balat 1974, 1976; Korniłłowicz-Kowalska and Kitowski 2009, 2013, 2017; Korniłłowicz-Kowalska et al. 2010, 2011, 2018; Jankowiak et al. 2019). The taxonomic and spatial distributions of the mycobiota in nests of wetland birds appear mainly affected by the properties of materials deposited in the nest constructions (Korniłłowicz-Kowalska et al. 2018). Most bird nests are built from various plant materials that are typically enriched by animal residues such as hairs, feathers, and insect exoskeletons, and that favour the development of various fungal groups, including cellulolytic and keratinolytic fungi (Korniłłowicz-Kowalska et al. 2010, 2018; AI Rubaiee et al. 2021). Bird nests also house potentially phytopathogenic and zoopathogenic fungi (Korniłłowicz-Kowalska and Kitowski 2013, 2017; Jankowiak et al. 2019). The predominance of ascomycetes appears to be a characteristic of open-cup bird nest mycobiota (Pugh 1965, 1966; Apinis and Pugh 1967; Otčenašek et al. 1967; Pugh and Evans 1970; Korniłłowicz-Kowalska and Kitowski 2009, 2017; Korniłłowicz-Kowalska et al. 2010, 2011, 2018). Interestingly, basidiomycetes are the dominant and co-dominant members of fungal communities in woodpeckers’ cavities excavated in dead or living trees (Jusino et al. 2015; Jankowiak et al. 2019; Pozzi et al. 2020).

As fungi are key producers of the enzymes required for cellulose and lignin decomposition (Schneider et al. 2012), measurements of the activity of extracellular enzymes involved in the circulation of nutrients that originate from organic compounds provide information about their biogeochemical cycles (Adamczyk et al. 2014). Several common hydrolytic enzymes contribute to the carbon (C) (β-D-cellobiosidase, β-glucosidase, β-xylosidase), nitrogen (N) (N-acetyl-β-glucominidase), phosphorus (P) (phosphatase), and sulphur (S) (arylosulphatase) cycles, and their main function is to degrade cellulose, hemicelluloses, and chitin (Parvin et al. 2018). Substrate availability and nutrient limitation are two strong drivers of enzyme activity in soil, and consequently of the C, N, and P cycles (Stock et al. 2019). Therefore, enzyme activity has been proposed as a quality indicator because it indicates changes in organic matter status and its turnover (Błońska et al. 2017). The decomposition of organic matter depends on substance properties and accessibility of microorganisms and their enzymes (Pająk et al. 2016).

The white stork *Ciconia ciconia* is an iconic species from European farmlands that builds one of the largest and heaviest nests among modern birds, which are used for many years. The nests are built of organic material such as hay, straw, manure, twigs, and branches at different stages of decomposition and as well as mineral particles (sand grains) and showed considerable variation in size (range: 80–230 cm in diameter; range: 10–140 cm in height) and weight 70–1348 kg (Zbyryt et al. 2021). In addition, the substrate composition of nests may change when parents incubate eggs and feed offspring; during subsequent breeding events, nests are enriched with excrement, pellets, feathers, eggshells, and food remains of invertebrate prey mainly insects and earthworms and small vertebrates mainly mammals. Large birds created a microhabitat in their nests which was favourable to nest-mediated seed dispersal (Dylewski et al. 2021). Recently, Błońska et al. (2021a) investigated the biological and physicochemical properties of white stork nests from north-east Poland and found that deposited materials gradually form soils over the years, with distinguishable layers possessing different physicochemical characteristics and biochemical activities. The results of this study indicated that the material contained in the nests could be characterised as lignic histosols ornithic. However, to the best of our knowledge, there are no reports that link the diversity of fungi in the open-cup nests of large-bodied birds to biological properties of stocked materials.

In our study, we hypothesised that large nest constructions are a specific microhabitat, characterised by diverse chemical properties that affect the enzyme activity and diversity of the fungal community. We also predicted the C/N/P ratio of material from large nests to be similar to that known in soils. C, N, and P are commonly considered major drivers of the enzyme activities and fungal diversity in soil. However, no studies have been conducted so far to test such relationships in material deposited in nests of large birds. As we expected considerable variation in the chemical properties of deposited material, we predicted these characteristics to significantly influence the enzyme activity, diversity of the culturable mycobiota associated with large-bodied birds.

## Materials and methods

### Study sites

The investigation was carried out in north-eastern Poland, in the Podlasie province, where materials from nineteen white stork nests were selected for biochemical examination and fungi cultivation. As the collection of material led to the destruction or serious damaging of the nest structure, the number and mass of the sample material are limited due to legal restrictions. The research covered nests used by white storks for several decades, although exact age of each nest is not known. Samples for analysis were collected from occupied nests after the breeding season. Nest samples were taken from a special crane raised several meters above the ground. Samples were collected from each nest to determine their chemical and biochemical properties. Further analyses were carried out using aggregate samples, homogenised from 5 smaller sub-samples from different places of nest so that all parts of the nest were represented (Carter and Gregorich 2008). The goal of sampling was to produce a sample that was representative for materials deposited in a given nest. The samples from each nest were placed in a plastic container and mixed. The containers and tools used to collect samples from the nests were washed with water and ethanol (> 99%, w/w) for disinfection before each sampling event. The samples were immediately transported to the laboratory and stored in the dark at 4 °C for subsequent analysis.

### Laboratory analysis

To determine their basic properties, 19 aggregate samples were dried to air dry condition and sieved (< 2-mm mesh) to remove artefacts (twine, plastic). Each sample was mixed in distilled water (1:5 w/w) and its pH was determined using the potentiometric method. The total N, organic C, and S contents were measured using a LECO CNS True Mac Analyzer (Leco, St. Joseph, MI, USA). Following mineralisation in a mixture of concentrated nitric acid and perchloric acid at a ratio of 2:1 v/v, the Ca, Na, Mg, K, P, Cd, Co, Cr, Cu, Fe, Mn, Ni, Pb, and Zn contents were determined using ICP-OES (Thermo iCAP 6500 DUO, Thermo Fisher Scientific, Cambridge, UK). C/N, C/P, and N/P ratios were calculated on a molecular basis (Piaszczyk et al. 2019).

Enzyme activities were determined using fluorogenic substrates (Pritsch et al. 2004; Sanaullah et al. 2016). Six fluorogenic enzyme substrates based on 4-methylumbelliferone (MUB) were used: MUB-β-D-cellobioside for β-D-cellobiosidase (CB), MUB-β-D-xylopyranoside for β-xylosidase (XYL), MUB-N-acetyl-β-D-glucosaminide for N-acetyl-β-D-glucosaminidase (NAG), MUB-β-D glucopyranoside for β-glucosidase (BG), MUB-phosphate for phosphatase (PH), and MUB-sulphate potassium salt for arylsulphatase (SP) (Turner 2010). We mixed 2.75 g of material from nests with 92 mL of universal buffer (pH 6.0). The soil suspension was then transferred into wells on a microwell plate, which contained the substrate and modified universal buffer. To measure fluorescence, the nest material suspension was first incubated for 1.5 h at 35 °C in 96-well microplates (Puregrade, Germany). The fluorescence was then determined using a multi-mode plate reader (SpectraMax), with excitation set at 355 nm and emission set at 460 nm. Analyses of chemical properties and enzymes activity were performed in triplicate.

### Fungal isolation and identification

Based on chemical properties of material from nests such as pH and carbon content, 11 nests were selected for fungal identification (Table 1). Each sample (50 g) in a state of natural moisture was stored in sterile plastic containers at 5 °C for 2 days prior to the isolation procedures. Culturable fungi were isolated using soil dilutions. A soil sample (10 g) was suspended in 90 mL of sterile distilled water and thoroughly mixed for 3 min to obtain a 10^−1^ suspension. Serial dilutions 10^−2^, 10^−3^, 10^−4^, and 10^−5^ were then prepared from this suspension. The methodology of the isolation procedures was previously described by Błońska et al. (2021a). Fungal taxa identified in nests of white stork and their frequencies (%) were detailed presented by Błońska et al. (2021a).

**Table 1:** Basic chemical properties of the material obtained from the nests of white stork *Ciconia ciconia*

Nest number	Symbol	pH in H_2_O	N	C	S	Ca	Cd	Co	Cr	Cu	Fe	K	Mg	Mn	Na	P	Pb	Zn
1	**SZ1**	4.30	13.5	171.9	2.9	3835.5	0.3	2.4	4.0	27.8	10160.0	2573.5	2457.5	162.6	403.4	1665.5	13.3	79.7
2	**SZ2**	4.55	11.9	128.6	2.3	4939.5	0.3	2.1	4.0	24.5	9824.0	3245.0	2247.0	150.8	577.7	2729.5	12.1	67.6
3	**M1**	3.97	19.2	180.1	4.1	6425.0	0.9	2.4	5.0	26.0	9221.5	2794.0	1953.5	181.6	504.4	3217.0	10.0	149.0
4	**M2**	4.61	24.6	225.8	6.2	14,445.0	1.6	2.6	2.0	32.3	9961.0	6202.5	3622.5	206.3	1687.5	7194.0	11.1	268.0
5	**GS1**	4.15	23.8	255.6	3.9	4166.5	0.3	1.3	1.4	34.2	9133.5	1787.0	1100.5	102.2	555.8	1956.5	8.4	70.9
6	**GS2**	3.67	21.8	266.5	3.6	4201.0	0.2	1.1	1.5	30.1	12,440.0	1163.5	1307.5	109.4	255.1	2116.0	10.4	60.6
7	**CZ**	5.49	17.4	180.6	3.3	7856.0	0.6	2.1	7.8	27.9	18,925.0	2786.5	2911.0	239.4	370.6	3958.0	13.1	128.7
8	**D23**	5.38	13.8	136.1	3.6	12,625.0	0.9	2.5	4.1	23.4	11,290.0	4196.5	3898.5	209.8	873.8	3684.5	11.2	126.5
9	**D37**	4.10	16.4	178.8	2.6	3968.0	0.2	1.3	0.0	25.0	6102.0	2319.0	2183.0	80.2	527.1	1106.5	6.6	43.6
10	**D2**	6.15	7.7	78.9	1.2	22,805.0	0.4	3.0	8.3	24.6	14,825.0	4839.5	11325.0	221.5	661.4	2399.0	16.5	92.4
11	**ŁA**	5.26	12.8	154.0	2.4	5928.5	0.3	1.6	5.1	27.7	15,715.0	2071.0	2040.0	172.5	259.3	2734.5	11.9	84.0
12	**BO**	4.16	22.1	197.1	4.3	6031.5	0.5	1.3	0.5	36.7	7078.5	2575.5	1843.0	105.2	550.5	2524.0	13.4	98.5
13	**DK**	4.17	22.0	222.1	4.5	3958.0	0.4	2.4	3.2	50.2	10,150.0	2944.0	2235.5	81.9	544.3	1613.0	11.0	90.2
14	**KO**	6.30	25.0	230.3	4.5	12,635.0	0.3	1.6	3.8	28.1	14,195.0	7797.0	4692.0	751.9	1155.5	4261.5	12.4	88.9
15	**ŁS**	4.38	9.0	114.4	1.9	3401.0	0.2	3.5	2.3	24.2	7814.5	2014.0	1316.5	74.0	375.8	1310.5	26.6	74.5
16	**P1**	3.73	22.3	251.1	4.2	3427.0	0.5	1.1	0.0	29.0	7041.5	954.5	863.4	107.1	287.6	1477.5	8.9	74.3
17	**P2**	4.06	14.3	169.6	2.5	4414.0	0.3	1.4	1.7	25.8	10,225.0	1332.5	1309.0	127.9	369.2	1662.0	11.7	64.5
18	**SU**	5.13	27.7	213.2	3.8	16,975.0	1.6	2.0	2.3	55.6	12,040.0	1169.5	1111.5	249.4	281.2	8156.5	10.0	340.1
19	**ZA**	4.19	18.6	183.3	3.4	4486.5	0.3	1.5	1.6	29.4	9511.0	2545.5	1992.5	165.4	604.7	1598.0	10.2	89.4
	**Mean:**	4.21	18.1	186.2	3.4	7711.7	0.5	2.0	3.1	30.7	10,823.8	2911.1	2653.1	184.1	570.8	2913.9	12.1	110.1
	**SD:**	0.78	5.7	50.2	1.1	5522.4	0.4	0.7	2.3	8.6	3258.0	1765.6	2331.8	148.2	350.0	1913.2	4.1	73.8

*N*, nitrogen content (g·kg^−1^); *C*, carbon content (g·kg^−1^); *S*, sulphur content (g·kg^−1^); content of micro- and macroelements (mg·kg^−1^)

Morphological identification was confirmed by sequencing the internal transcribed spacers 1 and 2 (ITS1-5.8S-ITS2). Altogether, 132 isolates were selected for molecular identification, and these were deposited in the culture collection of the Department of Forest Ecosystems Protection, University of Agriculture, Kraków, Poland (Błońska et al. 2021a). DNA was extracted using the Genomic Mini AX Plant Kit (A&A Biotechnology, Gdynia, Poland) according to the manufacturer’s protocol. The primers used were ITS 1F (Gardes and Bruns 1993) and ITS4 (White et al. 1990) for ITS1-5.8S-ITS2, Bt2a, and Bt2b (Glass and Donaldson 1995) for TUB2, and EF1 and EF2 (O’Donnell et al. 1998) or EF1–728 (Carbone and Kohn 1999) and TEF1rev (Kullnig-Gradinger et al. 2002) for TEF1-α.

### Statistical analysis

The Spearman’s rank correlation coefficients for the physical and chemical characteristics and enzyme activity of the 19 nest materials were calculated. Principal component analysis (PCA) was used to identify the interrelationships that existed among variables and to determine how suites of variables were related. The PCA method was used to evaluate the relationships between soil chemical properties, enzymatic activity, and fungal species composition in 11 nests. Differences with *p* < 0.05 were considered statistically significant. All statistical analyses were performed using the Statistica 13 software (2018).

For each nest sample, the number of colony-forming units (CFU) per gram of soil was determined based on the 10^−3^ dilution isolation results. The relative frequency (RF) was the ratio of the number of isolates of a certain taxon to the total number of isolates in each nest. Dominance (*Y*) was calculated as described by Du et al. (2020); when *Y* > 0.02, the genus was deemed dominant. The following ecological diversity measures used by Du et al. (2020) and characterised by Magurran (1988) were calculated for each nest: species richness index (SR), Margalef index (*D*′), Shannon–Wiener index (*H*′), Simpson diversity index (*D*_*s*_), Simpson dominant index (*λ*), probability of interspecific encounter index (PIE), and Pielou’s evenness index (*J*).

## Results

### Chemical properties and enzyme activity

The pH of the tested materials obtained from white stork nests ranged from 3.67 to 6.30 (Table 1). C, N, and S contents were high, yet diversified (78.9–266.5 g·kg^−1^, 7.7–27.7 g·kg^−1^ and 1.2–6.2 g·kg^−1^, respectively). P content ranged from 1598.0 to 8156.5 mg·kg^−1^. Ca, Mg, Na, and K contents were diverse, at 3401.0–22,805.0 mg·kg^−1^, 863.4–11,325.0 mg·kg^−1^, 255.1–1687.5 mg·kg^−1^ and 954.5–7797.0 mg·kg^−1^, respectively. The material was characterised by high Cd, Co, Cr, Mn, Pb, and Zn content (Table 1).

The studied material was also characterised by various enzyme activities (Table 2). CB activity ranged from 16.3 to 1261.1 nmol MUB^.^g^−1^ d.s.^.^h^−1^, BG activity from 307.7 to 3,261.7 nmol MUB^.^g^−1^ d.s.^.^h^−1^, NAG activity from 242.8 to 1772.4 nmol MUB^.^g^−1^ d.s.^.^h^−1^, and PH activity from 285.5 to 3222.8 nmol MUB^.^g^−1^ d.s.^.^h^−1^. We noted a lower activity for XYL and SP (33.7–399.2 nmol MUB^.^g^−1^ d.s.^.^h^−1^ and 0.0–43.3 nmol MUB^.^g^−1^ d.s.^.^h^−1^, respectively). The activity of most enzymes, except for SP, correlated with the C content (Table 3). The highest correlation with the content of C (0.83 and 0.82) was noted for the activity of CB and BG. The activity of CB, BG, and XYL correlated significantly with the content of N and S. A negative, significant correlation was noted between pH and the activity of CB and XYL (Table 3).

**Table 2:** Enzyme activities and C/N/P ratio of material from the nests of white stork *Ciconia ciconia*

Nest number	Symbol	CB	BG	NAG	XYL	SP	PH	C/N	C/P	N/P	C/N/P
1	**SZ1**	372.9	969.0	352.1	48.1	41.7	1000.0	14.8	266.2	18.0	266:18:1
2	**SZ2**	16.3	307.7	322.7	33.7	7.3	354.4	12.6	121.5	9.7	122:10:1
3	**M1**	569.2	1081.2	1668.1	292.0	19.1	613.1	10.9	144.4	13.2	144:13:1
4	**M2**	589.9	1597.4	708.2	145.3	42.3	424.7	10.7	80.9	7.5	81:8:1
5	**GS1**	824.2	2037.0	1397.4	267.3	17.2	1790.3	12.5	336.9	26.9	337:27:1
6	**GS2**	1261.1	2035.0	1391.3	388.9	32.7	851.3	14.2	324.8	22.8	325:23:1
7	**CZ**	373.6	1219.6	1608.3	105.0	33.7	895.6	12.1	117.7	9.7	118:10:1
8	**D23**	164.2	688.3	745.6	84.4	8.1	285.5	11.5	95.3	8.3	95:8:1
9	**D37**	226.2	1083.5	983.1	53.2	5.4	764.7	12.7	416.7	32.7	417:33:1
10	**D2**	65.2	334.0	384.4	40.0	4.3	140.8	11.9	84.8	7.1	85:7:1
11	**ŁA**	394.5	1198.1	1366.9	197.5	29.5	1086.3	14.0	145.2	10.4	145:10:1
12	**BO**	496.7	1359.5	775.6	118.4	0.0	833.8	10.4	201.4	19.4	201:19:1
13	**DK**	535.1	1639.9	1365.2	103.1	43.4	3222.8	11.8	355.1	30.2	355:30:1
14	**KO**	661.2	2436.3	1772.4	172.1	0.0	1765.6	10.7	139.4	13.0	139:13:1
15	**ŁS**	83.2	528.3	277.0	60.7	6.9	620.8	14.8	225.1	15.3	225:15:1
16	**P1**	1088.4	3261.7	928.5	399.2	11.0	1467.6	13.1	438.3	33.4	438:33:1
17	**P2**	382.9	1472.7	580.7	114.4	24.6	1623.1	13.8	263.2	19.0	263:19:1
18	**SU**	72.9	463.2	242.8	53.2	6.0	544.3	9.0	67.4	7.5	67:8:1
19	**ZA**	631.2	1700.5	867.4	173.8	0.0	1692.7	11.5	295.8	25.7	296:26:1
	**Mean:**	463.62	1337.5	933.6	150.0	17.7	1051.4	12.3	216.9	17.4	217:17:1
	**SD:**	340.48	760.2	508.9	113,0	15.4	738.2	1.6	118.7	9.0	

Enzyme activities (nmol MUB^.^g^−1^ d.s.^.^h^−1^); *CB*, β-D-cellobiosidase; *XYL*, β-xylosidase; *NAG*, N-acetyl-β-D-glucosaminidase; *BG*, β-glucosidase; PH, phosphatase; *SP*, arylsulphatase

**Table 3:** Spearman’s rank correlation coefficients between enzyme activities and carbon (C), nitrogen (N), phosphorus (P), sulphur (S) content and C/N, C/P and N/P ratios, and pH found in the material obtained from the nests of white stork *Ciconia ciconia*. Bold, significant terms at *p* < 0.05

	C	N	P	S	pH
CB	**0.83**	**0.63**	− 0.09	**0.65**	** − 0.48**
BG	**0.82**	**0.63**	− 0.18	**0.62**	− 0.39
NAG	**0.52**	0.36	0.11	0.43	− 0.16
XYL	**0.66**	**0.49**	− 0.02	**0.50**	** − 0.48**
SP	0.17	0.05	0.08	0.13	− 0.18
PH	**0.52**	0.35	− 0.37	0.31	0.29

*CB*, β-D-cellobiosidase; *XYL*, β-xylosidase; *NAG*, N-acetyl-β-D-glucosaminidase; *BG*, β-glucosidase; *PH*, phosphatase; *SP*, arylosulphatase

The nest material contained a C/N ratio of approximately 10 (in the range of 9.0 to 14.8). The mean C/P ratio was 216.9, with values ranging from 67.4 to 438.3. In the case of the N/P ratio, low values in the range of 7.1–33.4 were recorded. The C/N/P ratio in the materials from the nests was lower, and ranged from 67/8/1 to 438/33/1. There was a strong positive correlation between the C and N concentrations in the materials (*r* = 0.89) (Table 4). The concentrations of N and P correlated with the C/N ratio (*r* =  − 0.60). Positive correlations between C/N and C/P and between N/P and C/P were observed (*r* = 0.51 and *r* = 0.97, respectively) (Table 4).

**Table 4:** Spearman’s rank correlation coefficients between carbon (C), nitrogen (N), phosphorus (P) content and C/N, C/P and N/P ratios found in the material obtained from the nests of white stork *Ciconia ciconia*. Bold, significant terms at *p* < 0.05

	C	N	P	C/N	C/P	N/P
C						
N	**0.89**					
P	0.10	0.33				
C/N	** − **0.27	** − 0.60**	** − 0.60**			
C/P	0.34	0.05	** − 0.84**	**0.51**		
N/P	0.45	0.21	** − 0.78**	0.34	**0.97**	

### Diversity and composition of culturable fungi

Fungi from the white stork nests were from Ascomycota, Basidiomycota, and Mucoromycotina. The dominance values (*Y)* of *Penicillium* in Eurotiales, *Phialophora* in Chaetothyriales, *Pseudogymnoascus* in Thelebolales, *Mortierella* in Mortierellales, and *Trichoderma* in Hypocreales were 27.5752, 1.0704, 0.2952, 0.4290, and 0.1224, respectively. *Penicillium* spp. were the most dominant fungal genera among five nests (M1, M2, GS1, GS2, D37) and their *Y* values ranged from 25.0000 to 79.8595 (Table 5). The most dominant genus in the SZ1 nest was *Pseudogymnoascus* (*Y* = 48.1331), while Aspergillaceae were the most dominant in the D23 nest (*Y* = 18.5100). *Phialophora* was the dominant genus in four nests (SZ2, CZ, D37, and ŁA) and their *Y* values ranged from 3.3058 to 61.5927 (Table 5). The value of SR in most nest samples ranged from 11 to 21 (Table 6). The highest SR values were found in the M2 (SR = 28) and CZ (SR = 24) nests, while the lowest species richness values occurred in the ŁA (SR = 7) and GS1 (SR = 8) nests (Table 6). SR positively correlated with K and P contents. The Margalef index (*D*′) also showed a high correlation to Ca, Cd, Co, Mg, and Mn contents (Table 7).

**Table 5:** The dominance (*Y*) values of fungal genera or families obtained from the nests of white stork Ciconia ciconia*

Genus, family	SZ1	SZ2	M1	M2	GS1	GS2	CZ	D23	D37	D2	ŁA	Total
*Acaulium*	/	/	0.1238	0.0772	/	0.0240	/	0.4867	/	1.7243	/	0.0200
*Alternaria*	/	/	/	/	/	/	0.0080	/	/	1.7243	/	0.0031
*Aspergillus*	/	/	/	0.0123	/	0.0060	/	0.0541	0.0317	/	/	0.0014
Aspergillaceae	/	1.1718	/	1.4938	/	2.6501	0.0179	18.5100	/	/	/	0.1498
*Botryotrichum*	0.0366	/	/	/	/	/	3.5156	/	/	/	/	0.0293
*Chaetomium*	1.3187	0.5208	0.0077	/	/	/	0.5760	/	/	0.6530	/	0.0603
*Dinemasporium*	/	/	/	/	/	/	/	0.3380	/	1.2346	/	0.0035
*Fusarium*	/	/	/	/	/	/	0.9646	0.0135	/	0.0918	/	0.0094
*Leuconeurospora*	/	/	0.6742	0.0123	/	1.3521	/	/	6.9351	/	/	0.1961
*Mortierella*	1.1080	6.1218	1.4456	0.0031	0.5474	/	/	/	/	/	/	0.4290
*Mucor*	/	/	0.0009	0.6944	0.0001	/	/	/	/	0.0918	0.0160	0.0061
*Neosetophoma*	/	/	/	0.0031	/	/	0.0179	/	/	3.3058	/	0.0067
*Hawksworthiomyces* sp.	/	/	/	/	/	1.3521	/	/	/	/	/	0.0031
*Pascua*	/	/	/	/	/	/	/	/	0.1824	/	/	0.0020
*Penicillium*	0.0023	2.0831	40.8700	25.0000	79.8595	33.8021	5.1838	1.3521	26.9956	0.3673	2.7079	27.5752
*Phialophora*	0.0366	12.2861	0.1238	5.4444	/	/	8.9465	0.2163	0.0013	3.3058	61.5927	1.0704
*Pseudogymnoascus*	48.1331	/	0.0009	/	/	/	/	/	/	/	/	0.2952
*Sarocladium*	/	/	/	/	/	/	0.0717	/	/	0.1632	/	0.0014
*Scopulariopsis*	/	/	/	/	/	/	/	/	1.8288	/	/	0.0200
*Sporothrix*	/	/	0.4954	/	/	/	/	/	/	/	/	0.0080
*Trichoderma*	0.1854	0.5978	0.0009	/	0.0974	/	0.2412	5.9627	0.0317	0.0408	0.1442	0.1224

• Calculated for species where the total number of obtained colonies was ≥ 10; /, no determined

**Table 6:** Diversity indices for the assemblage of culturable fungi obtained from the nests of white stork *Ciconia ciconia*

Nest number	Symbol	Shannon–Wiener index (*H*′)	Simpson diversity index (D*s*)	Dominant index (*λ*)	Probability of interspecific encounter index (PIE)	Pielou index (*J*)	Margalef index (*D*′)	Species richness (SR)
1	SZ1	0.5163	0.5158	0.4841	0.5183	0.4957	4.2911	11
2	SZ2	0.9238	0.8149	0.1850	0.8189	0.6986	8.6356	21
3	M1	0.7827	0.7366	0.2633	0.7387	0.6235	6.7019	18
4	M2	0.9871	0.8434	0.1565	0.8478	0.6820	11.8367	28
5	GS1	0.5375	0.6272	0.3727	0.6279	0.5952	2.3833	8
6	GS2	0.8866	0.8297	0.1702	0.8361	0.7736	6.1209	14
7	CZ	1.0444	0.8591	0.1409	0.8628	0.7567	9.7308	24
8	D23	1.0795	0.8867	0.1132	0.8968	0.8297	9.7466	20
9	D37	0.7866	0.7939	0.2060	0.7967	0.6863	5.2891	14
10	D2	0.9538	0.8610	0.1389	0.8711	0.8322	6.7201	14
11	LA	0.3193	0.3598	0.6401	0.3644	0.3778	3.1618	7

**Table 7:** Spearman’s rank correlation coefficients between material properties and diversity indices of fungi obtained from in the nests of white stork *Ciconia ciconia*. Bold, significant terms at *p* < 0.05

	pH	N	C	S	Ca	Cd	Co	Cr	Cu	Fe	K	Mg	Mn	Na	P	Pb	Zn	C/N	C/P	N/P
Shannon–Wiener index (H0)	0.50	0.06	− 0.11	0.14	**0.68**	0.44	0.45	0.25	− 0.27	0.26	**0.65**	**0.68**	0.60	0.53	0.59	0.21	0.39	− 0.60	** − 0.70**	** − 0.64**
Simpson diversity index (Ds)	0.50	− 0.02	− 0.16	0.05	**0.71**	0.37	0.47	0.29	− 0.32	0.32	**0.61**	**0.69**	0.60	0.50	0.47	0.25	0.33	− 0.55	** − 0.67**	** − 0.64**
Dominant index (*λ*)	− 0.50	0.02	0.16	− 0.05	** − 0.71**	− 0.37	− 0.47	− 0.29	0.32	− 0.32	** − 0.61**	** − 0.69**	− 0.60	− 0.50	− 0.47	− 0.25	− 0.33	0.55	**0.67**	**0.64**
Probability of interspecific encounter index (PIE)	0.50	− 0.02	− 0.16	0.05	**0.71**	0.37	0.47	0.29	− 0.32	0.32	**0.61**	**0.69**	0.60	0.50	0.47	0.25	0.33	− 0.55	** − 0.67**	** − 0.64**
Pielou index (*J*)	0.34	− 0.18	− 0.23	− 0.19	0.52	0.06	0.27	0.25	− 0.43	0.29	0.39	0.52	0.39	0.31	0.18	0.23	0.01	− 0.30	− 0.44	− 0.45
Margalef index (D0)	0.45	0.05	− 0.16	0.21	**0.74**	**0.65**	**0.64**	0.32	− 0.29	0.23	**0.81**	**0.71**	**0.66**	0.55	**0.75**	0.26	0.58	** − 0.68**	** − 0.84**	** − 0.75**
Species richness (*S*)	0.25	0.22	0.03	0.30	0.55	0.55	0.46	0.16	− 0.15	0.04	**0.69**	0.50	0.50	0.46	**0.70**	0.10	0.50	** − 0.64**	** − 0.67**	− 0.55

The fungal diversity values varied widely among the sampled nests (Table 5). Fungal communities associated with SZ2, M2, GS2, CZ, D23, and D2 nests had the highest diversity (*H*′ and *D*_*s*_ ≥ 0.81), while the lowest diversity was found in the ŁA nest (*H*′ and *D*_*s*_ ≤ 0.31) (Table 6). PIE values were similar to *D*_*s*_ values, and the highest values were recorded in the following nests: D23, D2, CZ, M2, GS2, and SZ2. Fungi from D2 and D23 nests possessed the highest Pielou index (*J* = 0.83 and *J* = 0.83, respectively). The fungal community in ŁA and SZ1 nests showed the highest degree of ecological dominance with a *λ* value of 0.64 and 0.48, respectively. Conversely, the lowest *λ* value was detected in D23 and D2 nests (*λ* = 0.11 and *λ* = 0.14, respectively) (Table 6). The diversity indices statistically significant correlated with a content of Ca, K, and Mn in nests (Table 7).

**Fig. 1:**
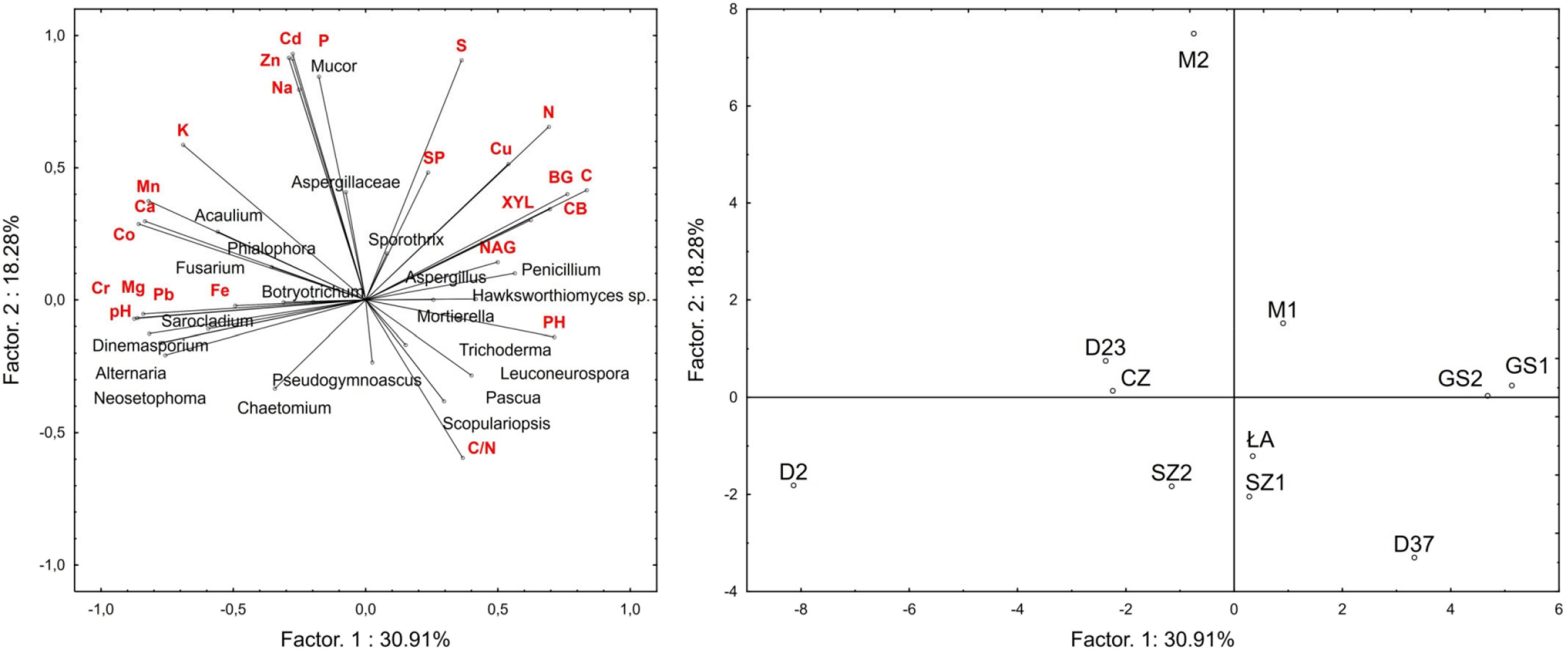
PCA diagram with projection of variables describing properties of material and fungal taxa present in the nests of white stork *Ciconia ciconia* on a plane of the first and second factors and the position of the samples in the plane formed by first two axes. The chemical properties, enzymes activity (for details of basic chemical properties and enzymes activity, see Tables 1 and 2, respectively), and fungal species composition (for list of culturable fungi, see Table 5) were used for diagram preparation

Two main PCA factors had a significant total impact (49.2%) on the variance of the variables. Factor 1 explained 30.9% of the variance of the examined properties, whereas factor 2 accounted for 18.3% of the variance (Fig. 1). The occurrence of *Penicillium*, *Leuconeurospora*, *Hawksworthiomyces* sp., *Mortierella*, *Aspergillus*, and *Scopulariopsis* was associated with high enzyme activity, as well as high C and N contents (Fig. 1). The higher C content and enzyme activity were noted in M1, GS1, GS2, and D37 nests (Fig. 1 and Table 1). Fungal communities associated with M1, GS1, GS2, and D37 nests were the most distant from the D2 nest, which was associated with high Pb, Mg, Cr, and Fe contents, as well as high pH (Fig. 1). The D2 nest was associated with *Alternaria*, *Dinemasporium*, *Neosetophoma*, and *Sarocladium* fungi (Fig. 1). In addition, the high content of K, Ca, Mn, and Co correlated with the presence of *Acaulium*, *Phialophora*, and *Fusarium* (Fig. 1). PCA analysis separated M2 nests mainly based on the prevalence of *Mucor* and Aspergiliaceae that were associated with high N, Cd, Zn, Na, and P contents and on the occurrence of *Scopulariopsis*, *Pseudogymnoascus*, and *Chaetomium*, that were associated with nests characterised by a low content of these elements (ŁA, SZ1, and SZ2 nests) (Fig. 1).

## Discussion

### Chemical properties of nests and their enzyme activities

We observed substantial differences in the chemical properties of materials deposited in the nests of white stork, which produced a high variability in enzymatic activity and composition of the fungal community. The nests were characterised by a high organic C and N contents, which led to the classification of white stork nests as lignic histosol ornithic (Błońska et al. 2021a). Birds transfer nutrients because the excreta of birds are rich in N and other nutrients such as P and Ca (Osono 2012). Organic matter is the basic parameter that influences soil biochemical activity by providing substrates for enzymatic reactions (Uzarowicz et al. 2020). Our findings confirmed the correlation between the extracellular enzyme activity involved in the C, N, and P cycles and the organic C content in white stork nests. The nests were also characterised by a high N content, which enabled the organic matter in the nests to decompose at a favourable rate. We recorded a suitable C/N ratio, which indicated the availability of N to soil microorganisms. The C/N ratio of material from white stork nests fluctuated to approximately 10. According to Robertson and Groffman (2015), microorganisms can easily obtain N during the decomposition of a relatively N-rich detritus (with a C/N ratio of approximately 20) because mineralisation outweighs the processes of N immobilisation and absorption. Organic C content in the nest material was the driver of all the tested hydrolytic enzyme activities that contributed to the C cycle (CB, BG, XYL), the N cycle (NAG), and the P cycle (PH). Additionally, β-D-cellobiosidase, β-glucosidase, and phosphatase activity significantly correlated with the pH of the tested material. pH influences the activity of soil enzymes by controlling ionisation caused by conformational changes in enzymes, substrate availability, and enzymatic cofactors (Błońska et al. 2021b). Kreyling et al. (2012) claimed that the organic matter in soil results from microbial degradation and the presence of nutrients such as N and P. According to WRB (2014) ornithogenic material contains at least 0.25% phosphorus pentoxide (P_2_O_5_) (in a 1% citric acid extract) and the tested nesting substrate met the criteria for onithogenic material (P_2_O_5_ content > 0.25%) (Błońska et al. 2021a). The obtained P value results meet the criteria defined in the WRB (2022) classification.

The samples collected from white stork nests were characterised by narrow C/N/P ratio ranging from 67/8/1 to 438/33/1, similar to that known for soils. Piaszczyk et al. (2019) noted that the C/N/P ratio for soils strongly influenced by decomposing deadwood ranged from 395/27/1 to 1592/81/1. The C/N/P ratio of the studied materials from nests was similar to that of mineral soils. The C/N/P ratio values obtained from the present study further confirm the possibility of soil formation in white stork nests. The C/N, C/P, and N/P ratios are good indicators of nutrient content and its potential availability (Piaszczyk et al. 2019). High C/N ratios indicate a slow decomposition of organic matter (Tian et al. 2010; Fazhu et al. 2015). The C/N ratio values obtained in the present study confirm the efficient decomposition of organic residues delivered to the nest. Negative correlations were noted between the C/N ratio and N and P, confirming that P was one of the key regulators of C and N circulation in the nest profile. Zhao et al. (2017) report that P can increase the labile fraction of C by increasing soil microbial activity. P prevails in nutrient limiting and controls the microbial community structure and succession (Knelman et al. 2014). According to Zhang et al. (2017), C, N, and P are the three main macroelements for biomass building, and the C/N/P ratio is therefore important to recognise the relationships between organisms and their environment.

### Culturable fungal diversity

In the present study, the 2,726 culturable fungi isolated from White Stork nests were divided into 82 taxa, 38 genera, 17 orders and 3 phyla, showing abundance and high species richness. This finding was consistent with those from studies conducted on other birds that build open-cup nests (Hubálek 2000; Korniłłowicz-Kowalska and Kitowski 2009, 2013, 2017; Korniłłowicz-Kowalska et al. 2010, 2011, 2018) and suggested that large birds’ nests that are occupied for long periods are a natural habitat for several fungal populations. For example, the nests of marsh harriers *Circus aeruginosus* yielded 63 fungal species belonging to 37 genera, among which *Asperigillus falvus*, *Asperigillus fumigatus*, *Penicillium* spp., and *Scopulariopsis brevicaulis* were the dominant ones (Korniłłowicz-Kowalska et al. 2018). Consistent with our results, most of the identified fungal species that dominate soil systems globally belong to phylum Ascomycota (Egidi et al. 2019). The abundance of fungi detected in nests could be due to a favourable C/N ratio in the nest material, which promoted the growth and activity of these microorganisms.

Our study revealed marked differences in the composition of fungal communities among white stork nests. The differences in the total number, species richness, and diversity indices of the communities that colonised the nests could be attributed to a variety of substrate characteristics, including the various chemical properties of the nest material and the nutrient availability, especially C and N sources. Despite the high content of organic C, N, and P in the nests, we observed considerable variation in the content of C, N, P, macro- and micro-nutrients. PCA revealed that the occurrence of *Penicillium* and *Aspergillus* fungi strongly correlated with the organic C content and high enzymes activities. These fungi are known for their high ability to secrete plant cell wall-degrading enzymes (Passos et al. 2018). The elevated organic C content in some nests could probably be linked to the higher proportion of plant debris used for its construction. The high activity levels of cellulolytic fungi may also result from raised N concentrations, as cellulase production is greatly influenced by the level and source of N in vitro (Lynd et al. 2002). Although we found no important effects of N content on fungal diversity, a previous study showed that the biomass and species richness of ectomycorrhizal fungi in pure culture increased under high N concentrations (Wilkinson et al. 2012). On the other hand, recent studies have indicated that the concentration of nitrogen fertiliser strongly modifies the composition but not the taxon richness of fungal communities in the soil (Paungfoo-Lonhienne et al. 2015). The abundance of ascomycetes is generally higher under high N concentrations and their growth is correlated with N availability (Fontaine et al. 2011).

Soil microbes play a crucial role in C, N, and P cycling in terrestrial ecosystems by mineralising organic matter (Zhou et al. 2018). Our findings show a strong relationship between fungal richness and diversity and the C/P and N/P ratios of the nest material. The stoichiometry of fungal biomass in response to the species taxonomy and ecological status, as well as to geographic and abiotic environmental factors, has been reported. According to Zhang and Elser (2017), Ascomycota fungi have lower C/P and N/P ratios than Basidiomycota fungi, which may explain their higher demand in relation to P. According to Camenzind et al. (2020), the stoichiometric data derived from nutrient gradient tests suggested a differential allocation of N and P in fungal mycelia during growth, indicating that fungi possess certain adaptive abilities when cultured on a substrate with different N and P contents. One of the major nutrients that influence microbial growth is P, and our study revealed that most of the material from white stork nests contained high amounts of P. Certain fungal species, especially saprotrophic ascomycetes (Zhang and Elser 2017) can accumulate P (Dietrich 1976; Ceci et al. 2018). However, zygomycetes (e.g. *Mucor* spp.) are also noted for their strong ability to mobilise P from different forms (Ye et al. 2015; Ceci et al. 2018; Domka et al. 2018). This strong affinity of *Mucor* spp. to survive in P-enriched environmental conditions was also confirmed by our results. In addition, unknown ascomycetes from the *Aspergillaceae* family showed an association with high P content. These results may reflect those from other studies where *Aspergillus*, *Penicillium*, and *Trichoderma* fungi are referred to as phosphate-solubilising fungi (Morales et al. 2011; Gaind 2016; Wang et al. 2018). It appears that fungi especially those with cellulolytic activity effectively uptake a large amount of organic P from the nest material. P is an important component that induces catabolic transformations and cellular synthesis (Griffin 1993). The species richness and diversity of culturable fungi were positively correlated with the level of Ca, K, and Mg in the nest material, with high levels significantly stimulating fungal growth. According to Jones (1965), Ca, K, and Mg cations promoted the growth of different fungi in vitro. High concentrations of these cations additionally reduced the negative impact of Na on fungal development Jones (1965). This is especially important given that the material from white stork nests sampled in our study contained relatively high amounts of Na.

The organic matter content in the soil is the primary factor that influences the abundance and diversity of fungi in Technosols (Stępniewska et al. 2020). Conversely, the concentration of trace elements plays a less important role in shaping the fungal variation. The results from the present study are consistent with those reported by Stępniewska et al. (2020). Despite the generally high concentrations of the elements, these were only recorded for Zn and Cu in several tested nests and did not reduce enzymatic activity and fungal diversity. Organic matter in the nest material probably reduced the toxicity of heavy metals in relation to enzymatic activity and fungi. Lasota et al. (2020) suggested that organic matter masks the negative impact of metals on the enzyme activity in the humus soil type. Soil organic matter influences metal binding and their impact on soil microorganisms (Wolińska et al. 2018). Nevertheless, the present study demonstrated that certain fungal species correlated with the concentration of heavy metals in the material of white stork nests. PCA revealed that *Mucor* spp. strongly correlated with Cd and Zn content, while Co, Cr, Pb, and Fe correlated with *Acaulium* sp., *Alternaria* sp., *Fusarium* sp., or *Sarocladium* sp. The high tolerance of *Mucor* sp. to elevated levels of Zn, Cd, and Pb was also recently described (Domka et al. 2018). Our study confirmed that fungi may occur very frequently in different environments with high concentrations of heavy metals (Lenart-Boroń and Wolny-Kładka 2015). The composition of culturable fungal communities detected in white stork nests resembled those found in different soil ecosystems (Frąc et al. 2018). The following fungal species with strong affinities to the soil environment were detected, among others, in the white stork nests: *Alternaria*, *Chaetomium*, *Fusarium*, *Penicillium*, *Sarocladium*, and *Trichoderma*. According to Egidi et al. (2019), these species represent well-known wind-dispersed fungal genera that are globally distributed and soil related. Our study also showed that the fungal community composition of white stork nests is more similar to that of agricultural ecosystems than that of forest ecosystems (Frąc et al. 2018). This composition corresponds to the natural habitats of the white stork, which are closely linked to the agricultural landscape (Butler et al. 2010).

## Conclusions

We observed notable differences in the chemical properties of materials deposited in the nests of the white stork, which produced a high variability in enzymatic activity. The results of the present study showed that the chemical properties of the material collected from white stork nests were correlated with the diversity of the culturable fungal community. Our findings revealed that fungi occurred frequently in materials with high levels of C, N, P, and trace elements. Our research confirmed low C/P and N/P ratio values in the studied nest materials, which indicate a high availability of P and, at the same time, favourable conditions for the development of fungi. Finally, we determined that C/N/P ratio was the main driver of fungal diversity in the studied nests.

## Data Availability

All data generated during the current study are included in this published article.

## References

[CR1] Adamczyk B, Kilpeläinen P, Kitunen VH, Smolander A (2014). Potential activities of enzymes involved in N, C, P and S cycling in boreal forest soil under different tree species. Pedobiologia.

[CR2] Al Rubaiee Z, Al Murayati H, Tobolka M, Tryjanowski P, Møller AP (2021). Not so black, not so white: differences in microorganisms load of contiguous feathers from white stork chicks. Curr Zool.

[CR3] Apinis AE, Pugh GJF (1967). Thermophilous fungi of birds’ nests. Mycopathology.

[CR4] Baggott GK, Graeme-Cook K, Deeming DC (2002). Microbiology of natural incubation. Avian incubation: behaviour, environment and evolution.

[CR5] Błońska E, Lasota J, Gruba P (2017). Enzymatic activity and stabilization of organic matter in soil with different detritus inputs. J Soil Sci Plant Nutr.

[CR6] Błońska E, Lasota J, Jankowiak R, Michalcewicz J, Wojas T, Zbyryt A, Ciach M (2021). Biological and physicochemical properties of the nests of White Stork Ciconia ciconia reveal soil entirely formed, modified and maintained by birds. Sci Total Environ.

[CR7] Błońska E, Piaszczyk W, Staszel K, Lasota J (2021). Enzymatic activity of soils and soil organic matter stabilization as an effect of components released from the decomposition of litter. Appl Soil Ecol.

[CR8] Butler SJ, Boccaccio L, Gregoryc RD, Vorisek P, Norris A (2010). Quantifying the impact of land use change to European farmland bird populations. Agric Ecos Environ.

[CR9] Camenzind T, Grenz KP, Lehmann J, Rillig MC (2020). Soil fungal mycelia have unexpectedly flexible stoichiometric C;N and C: P ratios. Ecol Lett.

[CR10] Carbone I, Kohn LM (1999). A method for designing primer sets for speciation studies filamentous ascomycetes. Mycologia.

[CR11] Carter MR, Gregorich EG (2008). Soil sampling and methods of analysis.

[CR12] Ceci A, Pinzari F, Russo F, Maggi O, Persiani AM (2018). Saprotrophic soil fungi to improve phosphorus solubilisation and release: in vitro abilities of several species. Ambio.

[CR13] Dietrich SM (1976). Presence of polyphosphate of low molecular weight in Zygomycetes. J Bacteriol.

[CR14] Domka A, Rozpądek P, Ważny R, Turnau K (2018). *Mucor* sp. – an endophte of Brassicaceae capable of surviving in toxic metal-rich sites. J Basic Microb.

[CR15] Du W, Yao Z, Li J, Sun Ch, Xia J, Wang B, Shi D, Ren L (2020). Diversity and antimicrobial activity of endophytic fungi isolated from Securinega suffruticosa in the Yellow River Delta. PLoS.

[CR16] Dylewski Ł, Dyderski MK, Maćkowiak Ł, Tobolka M (2021). Nests of the white stork as suitable microsites for the colonisation and establishment of ruderal plants in the agricultural landscape. Plant Ecol.

[CR17] Egidi E, Delgado-Baquerizo M, Plett JM, Wang J, Eldridge DJ, Bardgett RD (2019). A few Ascomycota taxa dominate soil fungal communities worldwide. Nat Commun.

[CR18] Fazhu Z, Jiao S, Chengjie R, Di K, Jian D, Xinhui H, Gaihe Y, Yongzhong F, Guangxin R (2015). Land use changes influences the soil C, N, and P stoichiometry under “Grain-to-green Program” in China. Sci Rep.

[CR19] Fontaine S, Henault C, Aamor A, Bdioui N, Bloor JMG, Maire V, Mary B (2011). Fungi mediate long term sequestration of carbon and nitrogen in soil through their priming effect. Soil Biol Biochem.

[CR20] Frąc M, Jezierska-Tys S, Takashi Y (2015). Occurrence, detection, and molecular and metabolic characterization of heat-resistant fungi in soils and plants and their risk to human health. Adv Agron.

[CR21] Frąc M, Hannul SE, Bełka M, Jędryczka M (2018). Fungal biodiversity and their role in soil health. Front Microbiol.

[CR22] Gaind S (2016). Phosphate dissolving fungi: mechanism and application in alleviation of salt stress in wheat. Microbiol Res.

[CR23] Gardes M, Bruns TD (1993). ITS primers with enhanced specificity for Basidiomycetes – application to the identification of mycorrhiza and rusts. Molecul Ecol.

[CR24] Glass NL, Donaldson GC (1995). Development of primer sets designed for use with the PCR to amplify conserved genes from filamentous ascomycetes. Appl Environ Microbio.

[CR25] Griffin DH (1993). Fungal physiology.

[CR26] Hubálek Z, Balat F (1974). The survival of microfungi in the nests of tree sparrow (Passer montanus L.) in the nest-boxes over the winter season. Mycopathol Mycol Appl.

[CR27] Hubálek Z, Balat F (1976). Seasonal distribution of keratinolytic fungi in the nests of tree sparrow (Passer montanus L.). Zentralblatt Für Bakteriologie, Mikrobiologie Und Hygiene.

[CR28] Hubalek Z (2000) Keratinophilic fungi associated with free living mammals and birds. In: R. K. S. Kushwaha, J. Guarro (eds). Biology of dermatophytes and other keratinophilic fungi. Rev Iber Micol 17: 104–108

[CR29] IUSS Working Group WRB (2022) World reference base for soil resources. International soil classification system for naming soils and creating legends for soil maps. 4th edition. International Union of Soil Sciences (IUSS), Vienna, Austria

[CR30] Jankowiak R, Ciach M, Bilański P, Linnakoski R (2019). Diversity of wood-inhabiting fungi in woodpecker nest cavities in southern Poland. Acta Mycol.

[CR31] Jones EBG (1965). The effect of cations on the growth of fungi. New Phytol.

[CR32] Jusino MA, Lindner DL, Banik MT, Walters JR (2015). Heart rot hotel: fungal communities in red cockaded woodpecker excavations. Fungal Ecol.

[CR33] Knelman JE, Schmidt SK, Lynch RC, Darcy JL, Castle SC, Cleveland CC, Nemergut DR (2014). Nutrient addition dramatically accelerates microbial community succession. PLoS One.

[CR34] Korniłłowicz-Kowalska T, Kitowski I (2009). Diversity of fungi in nest and pellets of Montagu’s harrier (Circus pygargus) from eastern Poland – importance chemical and ecological factors. Ecol Chem Eng.

[CR35] Korniłłowicz-Kowalska T, Kitowski I (2013). Aspergillus fumigatus and other thermophilic fungi in nests of wetland birds. Mycopathology.

[CR36] Korniłłowicz-Kowalska T, Kitowski I (2017). Nests of marsh harrier (Circus aeruginosus L.) as refuges of potentially phytopathogenic and zoopathogenic fungi. Saudi J Biol Sci.

[CR37] Korniłłowicz-Kowalska T, Kitowski I, Iglik H (2010). The occurrence of cellulolytic fungi and Fusarium in nests of Circus pygargus. Acta Mycol.

[CR38] Korniłłowicz-Kowalska T, Kitowski I, Iglik H (2011). Geophilic dermatophytes and other keratinophilic fungi in the nests of wetland birds. Acta Mycol.

[CR39] Korniłłowicz-Kowalska T, Kitowski I, Bohacz J, Kwiatkowska E (2018). Fungal frequency and diversity in the nests of wetland birds from Poland: relationships between birds, nest properties and inhabiting fungi. Avian Biol Res.

[CR40] Kreyling J, Peršoh D, Werner S, Benzenberg M, Wöllecke J (2012). Short-term impacts of soil freeze-thaw cycles on roots and root-associated fungi of Holcus lanatus and Calluna vulgaris. Plant Soil.

[CR41] Kullnig-Gradinger C, Szakacs G, Kubicek CP (2002). Phylogeny and evolution of the genus Trichoderma: a multigene approach. Mycol Res.

[CR42] Lasota J, Błońska E, Łyszczarz S, Tibbett M (2020). Forest humus type governs heavy metal accumulation in specific organic matter fractions. Water Air Soil Pollut.

[CR43] Lenart-Boroń A, Wolny-Koładka K (2015). Heavy metal concentration and the occurrence of selected microorganisms of steelworks area in Poland. Plant Soil Environ.

[CR44] Lynd LR, Weimer PJ, Van Zyl WH, Pretorius IS (2002). Microbial cellulose utilization: fundamentals and biotechnology. Microbiol Mol Biol.

[CR45] Magurran AE (1988). Ecological diversity and its measurement.

[CR46] Morales A, Alvear M, Valenzuela E, Castillo CE, Borie F (2011). Screening, evaluation and selection of phosphate-solubilising fungi as potential biofertiliser. J Soil Sci Plant Nutr.

[CR47] O'Donnell K, Kistler HC, Cigelnik E, Ploetz RC (1998). Multiple evolutionary origins of the fungus causing Panama disease of banana: concordant evidence from nuclear and mitochondrial gene genealogies. Proc Nat Acad Sci USA.

[CR48] Osono T, Young SS, Silvern SE (2012). Excess supply of nutrients, fungal community, and plant litter decomposition: a case study of avian-derived excreta deposition in conifer plantations. International Perspectives on Global Environmental Change.

[CR49] Otčenašek M, Hudec K, Hubalek Z, Dvorak I (1967). Keratinophilic fungi from the nests of birds in Czechoslovakia. Sabouraudia.

[CR50] Pająk M, Błońska E, Frąc M, Oszust K (2016). Functional diversity and microbial activity of forest soils that are heavily contaminated by lead and zinc. Water Air Soil Poll.

[CR51] Parvin S, Blagodatskaya E, Becker JN, Kuzyakov Y, Uddin S, Dorodnikov M (2018). Depth rather than microrelief controls microbial biomass and kinetics of C-, N-, P- and S-cycle enzymes in peatland. Geoderma.

[CR52] Passos DF, Pereira N, de Castro AM (2018). A comparative review of recent advances in cellulases production by Aspergillus, Penicillium and Trichoderma strains and their use for lignocellulose deconstruction. Curr Opin Green Substain Chem.

[CR53] Paungfoo-Lonhienne C, Yeoh YK, Kasinadhuni NRP, Lonhienne TGA, Robinson N, Hugenholtz P, Ragan MA, Schmidt S (2015). Nitrogen fertilizer dose alters fungal communities in sugarcane soil and rhizosphere. Sci Rep.

[CR54] Piaszczyk W, Błońska E, Lasota J, Lukac M (2019). A comparison of C:N: P stoichiometry in soil and deadwood at an advanced decomposition stage. CATENA.

[CR55] Pozzi C, Raichenberg M, Ojeda V (2020). Decay fungi associated with cavity excavation by a large South American woodpecker. For Pathol.

[CR56] Pritsch K, Raidl S, Marksteiner E, Blaschke H, Agerer R, Schloter M, Hartmann A (2004). A rapid and highly sensitive method for measuring enzyme activities in single mycorrhizal tips using 4- methylumbelliferone-labelled fluorogenic substrates in a microplate system. J Microbiol Meth.

[CR57] Pugh GIE (1965). Cellulolytic and keratinophilic fungi recorded on birds. Sabouraudia.

[CR58] Pugh GIE (1966). Associations between birds, nests, their pH and keratinophilic fungi. Sabouraudia.

[CR59] Pugh GIE, Evans MD (1970). Keratinophilic fungi associated with birds: I. Fungi isolated from feathers nests and soil. Trans Br Mycol Soc.

[CR60] Robertson GP, Groffman PM, Paul EA (2015). Nitrogen transformations. Soil microbiology, ecology and biochemistry.

[CR61] Rousk J, Bååth E (2011). Growth of saprotrophic fungi and bacteria in soil. FEMS Microbiol Ecol.

[CR62] Sanaullah M, Razavi BS, Blagodatskaya E, Kuzyakov Y (2016). Spatial distribution and catalytic mechanisms of β-glucosidase activity at the root-soil interface. Biol Fertil Soils.

[CR63] Schneider T, Keiblinger KM, Schmid E, Sterflinger-Gleixner K, Ellersdorfer G, Roschitzki B, Richter A, Eberl L, Zechmeister-Boltenstern S, Riedel K (2012). Who is who in litter decomposition? Metaproteomics reveals major microbial players and their biogeochemical functions. ISME J.

[CR64] Stępniewska H, Uzarowicz Ł, Błońska E, Kwasowski W, Słodczyk Z, Gałka D, Hebda A (2020). Fungal abundance and diversity as influenced by properties of Technosols developed from mine wastes containing iron sulphides: a case study from abandoned iron sulphide and uranium mine in Rudki, south-central Poland. Appl Soil Ecol.

[CR65] Stock SC, Köster M, Dippold MA, Nájera F, Matus F, Merino C, Boy J, Spelvogel S, Gorbushina A, Kuzyakov Y (2019). Environmental drivers and stoichiometric constraints on enzyme activities in soils from rhizosphere to continental scale. Geoderma.

[CR66] Tian HQ, Chen GS, Zhang C, Melillo JM, Hall CAS (2010). Pattern and variation of C/N/P ratios in China's soils: a synthesis of observational data. Biogeochemistry.

[CR67] Treseder KK, Lennon JT (2015). Fungal traits that drive ecosystem dynamics on land. Microbiol Mol Biol Rev.

[CR68] Turner BL (2010). Variation in pH optima of hydrolytic enzyme activities in tropical rain forest soils. Appl Environ Microb.

[CR69] Uzarowicz Ł, Wolińska A, Błońska E, Szafranek-Nakonieczna A, Kuźniar A, Słodczyk Z, Kwasowski W (2020). Technogenic soils (Technosols) developed from mine spoils containing Fe sulphides: microbiological activity as an indicator of soil development following land reclamation. Appl Soil Ecol.

[CR70] Wang X, Wang Ch, Sui J, Liu Z, Li Q, Ji Ch, Song X, Hu Y, Wang C, Sa R, Zhang J, Du J, Liu X (2018). Isolation and characterization of phosphofungi, and screening of their plant growth-promoting activities. AMB Express.

[CR71] White TJ, Bruns T, Lee S, Taylor J, Innis MA, Gelfand DH, Snisky JJ, White TJ (1990). Amplification and direct sequencing of fungal ribosomal RNA genes for phylogenetics. PCR protocols: a guide to methods and applications.

[CR72] Wilkinson A, Solan M, Alexander I, Johnson D (2012). Species richness and nitrogen supply regulate the productivity and respiration of ectomycorrhizal fungi in pure culture. Fungal Ecol.

[CR73] Wolińska A, Banach A, Szafranek-Nakonieczna A, Stępniewska Z, Błaszczyk M (2018). Easily degradable carbon - an indicator of microbial hotspots and soil degradation. Int Agrophys.

[CR74] WRB (2014) World Reference Base For Soil Resource. FAO, ISRIC and ISSS

[CR75] Ye Y, Gan J, Hu B (2015). Screening of phosphorus-accumulating fungi and their potential for phosphorus removal from waste streams. Appl Biochem Biotechnol.

[CR76] Zbyryt A, Dylewski Ł, Neubauer G (2021). Mass of white stork nests predicted from their size: online calculator and implications for conservation. J Nature Conserv.

[CR77] Zhang J, Elser JJ (2017). Carbon: nitrogen: phosphorus stoichiometry in fungi: A meta-analysis. Front Microbiol.

[CR78] Zhang Z, Xue Z, Lyu X, Tong S, Jiang M (2017). Scaling of soil carbon, nitrogen, phosphorus and C/N/P ratio patterns in peatlands of China. Chinese Geogr Sci.

[CR79] Zhao H, Sun B, Lu F, Wang X, Zhuang T, Zhang G, Ouyang Z (2017). Roles of nitrogen, phosphorous and potassium fertilizers in carbon sequestration in a Chinese agricultural ecosystems. Clim Chang.

[CR80] Zhou X, Sun H, Pumpanen J, Sietiö OM, Heinonsalo J, Köster K, Berninger F (2018). The impact of wildfire on microbial C:N: P stoichiometry and the fungal-to-bacterial ration in permafrost soil. Biogeochemistry.

